# Inhibitory Effect of DNase–Chitosan–Nisin Nanoparticles on Cell Viability, Motility, and Spatial Structures of *Listeria monocytogenes* Biofilms

**DOI:** 10.3390/foods13223544

**Published:** 2024-11-06

**Authors:** Xinyi Pang, Xueying Du, Xin Hu, Zeyin Feng, Jing Sun, Xiangfei Li, Yingjian Lu

**Affiliations:** College of Food Science and Engineering, Nanjing University of Finance and Economics, Nanjing 210023, China; pangxinyi@nufe.edu.cn (X.P.); xueydu@163.com (X.D.); 15920834382@163.com (X.H.); fengzeyin2000@163.com (Z.F.); jingsun@nufe.edu.cn (J.S.); xiangfeili@nufe.edu.cn (X.L.)

**Keywords:** nanoparticles, *Listeria monocytogenes*, biofilms, nisin, DNase I

## Abstract

*Listeria monocytogenes* biofilm contamination on food contact surfaces is a major concern for the food industry. Nanoparticle encapsulation appears as a novel strategy for food surface disinfection to prevent biofilm formation. Chitosan nanoparticles loaded with nisin and DNase I (DNase-CS-N) have been constructed to exhibit antimicrobial activity against *L. monocytogenes*. This study aimed to investigate their ability to inhibit *L. monocytogenes* biofilm formation and eliminate preformed biofilms on food contact surfaces (polystyrene, polyurethane, and stainless steel). DNase-CS-N could decrease 99% and 99.5% biofilm cell numbers at 1/2 MIC and MIC, respectively. At sub-MICs, DNase-CS-N could reduce cell motility (swimming and swarming) and slime production of *L. monocytogenes*. In terms of effect on biofilm elimination, DNase-CS-N at the concentration of 4 MIC led to 3–4 log reduction in biofilm cells in preformed biofilms, performing higher efficiency compared with other treatments (CSNPs, CS-N). Furthermore, the three-dimensional structure of *L. monocytogenes* biofilms was severely disrupted after DNase-CS-N treatment, with bacterial cells scattered on the surface. The morphology of biofilm cells was also greatly damaged with wrinkled surfaces, disrupted cell membranes, and leakage of intracellular nucleic acids and proteins. These results indicate the potential applicability of DNase-CS-N for inhibiting and eliminating *L. monocytogenes* biofilms on food contact surfaces.

## 1. Introduction

*Listeria monocytogenes* is known as the etiological agent of listeriosis, a life-threatening foodborne disease with high fatality of about 40–60% [1]. *L. monocytogenes* is ubiquitous in natural environments and has a propensity to adhere to food contact surfaces, including stainless steel (SS), polypropylene, and glass at different temperatures [2]. Sudagidan et al. [3] revealed that 21 strains of *L. monocytogenes* isolated from smoked salmon and fish contact surfaces could all form dense biofilms on glass surfaces. Bonsaglia et al. [4] analyzed the ability of 32 *L. monocytogenes* strains isolated from different foods (milk and vegetables) and food-processing environments to form biofilms on three different surfaces (glass, polystyrene, and SS). The results showed that all strains produced biofilms at at least one of the temperatures and materials tested. *L. monocytogenes* biofilms might persist in food processing plants for months to decades [5,6]. One of the widely known mechanisms for *L. monocytogenes* to persist in food processing facilities is its ability to form biofilms to withstand cleaning and disinfection procedures. Being embedded in a matrix of extracellular polymeric substances (EPS), bacterial cells within biofilms can be protected from various unfavorable environments [7]. The biofilm matrix of *L. monocytogenes* mainly comprises extracellular DNA, protein, and exopolysaccharides. Many recent studies are focusing on developing novel intervention/prevention strategies to control *L. monocytogenes* biofilms in the food industry [8,9].

Plant extracts and essential oils to inhibit *L. monocytogenes* biofilms have attracted and encouraged great research interest. Pinto et al. [10] reported that oregano essential oil vapor reduces viable cells of *L. monocytogenes* on food-contact surfaces below the detection limit (200 CFU coupon^−1^). The research by Somrani et al. [11] showed that garlic essential oil, onion essential oil, and cinnamon essential oil could inhibit initial cell attachment of *L. monocytogenes* and eradicate the preformed biofilms. The inhibition of *L. monocytogenes* preformed biofilm by garlic essential oil, onion essential oil, and cinnamon essential oil was 61%, 53%, and 68%, respectively.

Nisin is a bacteriocin produced by *Lactococcus lactis* that exhibits great antibacterial activity against a wide range of Gram-positive foodborne pathogens. The anti-listeria activity of nisin has been demonstrated in many food products, and its mechanisms have also been elucidated [12]. However, the antibacterial activity of nisin against biofilm cells compared to planktonic cells is greatly compromised due to the limited penetration of nisin into biofilm matrix and the interference of biofilm EPS [13].

To solve this problem, nisin has been combined with other antimicrobial hurdles to enhance the ability to inhibit biofilm formation and eradicate mature *L. monocytogenes* biofilms. Hossain et al. [14] showed that nisin was not able to inhibit biofilm formation to an acceptable level when it acted alone, but the inhibitory effect remarkably increased when it was used in combination with essential oils (thymol, eugenol). Wu et al. [15] combined nisin with sesamol effectively prevent *L. monocytogenes* biofilm formation on SS surfaces within 48 h, and their combination successfully eradicated the established *L. monocytogenes* biofilms within 4 h treatment. Zhou et al. [16] used nisin in conjunction with a free fatty acid (lauric acid/N-tridecanoic acid) to treat the *L. monocytogenes* biofilms. No re-growth of *L. monocytogenes* was observed during co-treatment with nisin and fatty acids over a 24 h incubation, in contrast to nisin treatment alone. Although great efforts above have improved the efficiency of nisin to control *L. monocytogenes* biofilms, the low stability and fast release of pure nisin still limit its application in food processing environments [17].

In recent years, nanoparticles (NPs) with unique physical and chemical properties, great bactericidal activity, and specific mechanisms have attracted much attention targeting bacterial biofilms [18]. Chitosan is one of the widely used polysaccharides for the fabrication of NPs due to being non-toxic, biodegradable, and biocompatible [19]. In addition, chitosan possesses both antimicrobial properties and film-forming capabilities, making it a suitable candidate for use in food packaging and preservation. Research has encapsulated nisin within chitosan nanoparticles (CSNPs), resulting in an enhanced antimicrobial effect against *L. monocytogenes* compared to its unencapsulated, free-form [20,21,22]. However, few studies have evaluated the antibiofilm activity of CSNPs. For instance, Ammar et al. [23] investigated the antibiofilm activity of CSNPs loaded with carvacrol against *L. monocytogenes*. The results showed that the biofilm inhibition rate reached 35.79%, 73.37%, and 77.76% when the concentration of nanoparticles was 0.5 MIC, MIC, and 2 MIC, respectively.

The main reason for the increased resistance of biofilm cells compared to their planktonic counterparts is mainly the EPS, which functions as a physical scaffold to protect the cells within the biofilm matrix. Thus, the biofilm matrix eDNA has been considered an important target for the treatment of *Pseudomonas aeruginosa* and *S. aureus* biofilm infections clinically [24,25]. In our previous study, we constructed DNase I functionalized CSNPs with an MIC of 1.28 mg/mL, and it reduced 3 log CFU/cm^2^ of *L. monocytogenes* biofilm cells on polyurethane (PU) surfaces [26]. However, the antibiofilm activity of nanoparticles against *L. monocytogenes* on food-contact surfaces is still not known. Thus, in this study, the efficacy of nisin-loaded CSNPs functionalized with DNase I to inhibit biofilm cell viability and motility and disrupt mature biofilms on different food-contact surfaces was evaluated. Moreover, the effect of NPs on the biofilm formation of *L. monocytogenes* at different stages, including cell motility, spatial organization, and the physiological state of adhered cells, was also investigated to decipher possible mechanisms for biofilm inactivation.

## 2. Materials and Methods

### 2.1. Bacterial Strains and Culture Conditions

*L. monocytogenes* FSIS 57034 (serotype 1/2c) obtained from Nanjing Agricultural University was used in this study. The bacteria were activated by inoculating 100 μL from the stock to 10 mL of tryptic soy broth (TSB, Qingdao Nissu Biotechnologies Co., Ltd., Qingdao, China) and were separately activated twice for 24 h at 37 °C. Overnight cultures were harvested by centrifugation at 5000× *g* for 5 min at 4 °C and then resuspended in 0.9% NaCl to achieve a concentration of 10^9^ CFU/mL as working cultures.

### 2.2. Preparation of NPs

The preparation method of three NPs, namely CSNPs, nisin-loaded CSNPs (CS-N), and CS-N grafted with DNase I (DNase-CS-N), has been mentioned in our previous study [26]. Briefly, CS-N was prepared by the addition of a mixture of nisin and TPP dropwise to CS solution (CS:TPP = 5:1) under magnetic stirring (LICHEN). The CS-N was collected by centrifugation at 13,000 rpm (Thermo Fisher, Waltham, MA, USA), 4 °C for 30 min, and then dispersed in distilled water. CSNPs were obtained by replacing nisin in the above method with an equal amount of ultrapure water. The mixture of EDC and NHS with the final concentration of 0.1 M was added dropwise to the CS-N solution, followed by the addition of DNase I (200 μg/mL) solution and stirring for 30 min. DNase-CS-N was obtained by dissolving in ultrapure water again after centrifugation (conditions as above).

### 2.3. Quantification of Viable Cell Counts of L. monocytogenes Biofilms

Polystyrene (PS), PU, and SS coupons (1 cm × 1 cm × 0.1 cm) were used to simulate contact surfaces in food processing environments. Prior to use, the coupons were first sonicated in a detergent solution for 30 min, followed by sonication in 70% (*v*/*v*) ethanol for 15 min [27]. After rinsing with distilled water, the coupons were air-dried and subsequently autoclaved at 121 °C for 15 min.

The MIC experiment has been previously conducted and reported in our previous study. The results indicate that MIC is about 1.28 mg/mL for all the NPs [26]. For biofilm cell counts in inhibitory concentration, 500 μL of *L. monocytogenes* working cultures (10^5^ CFU/mL) and 500 μL of NPs added to achieve the final concentrations (2 MIC, MIC, 1/2 MIC, and 1/4 MIC) were simultaneously mixed in a 24-well plate (Labgic Technology Co., Ltd., Beijing, China) containing one coupon per well. The plates were incubated statically at 37 °C for 48 h for biofilm development. Following incubation, the coupons were washed three times with physiological saline and then transferred into centrifuge tubes containing 5 mL of physiological saline. The tubes were subjected to ultrasonication at 40 kW for 3 min, followed by vigorous vortexing for 30 s. The detached *L. monocytogenes* biofilm cell suspension was diluted and spread onto TSA plates at appropriate dilutions. The TSA plates were incubated at 37 °C for 24 h, followed by colony counting.

For the biofilm elimination assay, the coupons were first immersed into a fresh TSB medium containing *L. monocytogenes* (10^5^ CFU/mL) at 37 °C for 48 h. After being rinsed as mentioned above, the coupons were submerged in PBS (Beijing Solarbio Science & Technology Co., Ltd., Beijing, China) solution containing NPs at different concentrations (4 MIC, 2 MIC, and MIC) for 12 h. Then, the coupons were subjected to sonication and vortex for biofilm detachment, and the plate counting method was used for the measurement of the biofilm cell population, as mentioned above.

### 2.4. Cell Motility Assay

A working culture of *L. monocytogenes* suspension (10^5^ CFU/mL) was added to the test tube, followed by the addition of DNase-CS-N to achieve the final concentration of MIC, 1/2 MIC, and 1/4 MIC. The tubes were incubated at 30 °C for 24 h. Subsequently, an inoculation needle was dipped into the bacterial suspension and used to shallowly stab the center of TSA plates containing 0.3% and 0.5% agar for swimming and swarming studies, respectively. The plates were incubated upright at 30 °C for 24 h. The diameter of the motility zones was measured using a vernier caliper.

Measurement of bacterial swimming and swarming motility using agar concentrations of 0.3% and 0.5%, respectively, is based on the ability of the bacteria to pass through the solidifying medium with different viscosities. Agar at a concentration of 0.3% provides an environment that approximates a liquid, allowing bacteria to swim freely [28]. This type of motility refers to the movement of individual bacteria. In such an environment, bacteria rely on the rotation of their flagella to propel themselves in particular directions [29]. In contrast, swarm movement is characterized by the multicellular movement of bacteria that migrate above a solid substrate in the form of tightly bound cells. This form of motility also depends on flagella but typically involves additional factors, such as surface tension and the production of extracellular polysaccharides [30].

### 2.5. Slime Layer Production

The production of slime by *L. monocytogenes* was assessed using Congo Red Agar (CRA; Beijing Solarbio Science & Technology Co., Ltd., Beijing, China) plates according to Piercey et al. [31] with some modifications. After autoclaving the TSA medium, the temperature was cooled to 55 °C, and Congo Red dye (0.8 g/L) and sucrose (36 g/L) were added to the TSA. The mixture was then poured into plates and allowed to solidify for future use. Strains treated and untreated with DNase-CS-N, as obtained from the method described in Section 2.4, were streaked onto the CRA plates using an inoculating loop. The plates were incubated at 37 °C for 48 h to observe slime production. The method is based on the ability of Congo red indicator to bind to the polysaccharide matrix produced by bacteria [32]. When bacteria grow on CRA plates and produce slime (including polysaccharide substrates), Congo red binds to these polysaccharide substrates, resulting in color changes. Specifically, the color of Congo red fades from red to black after incubation. Therefore, strains producing slime (characterized by black colonies on red agar) can be identified by observing CRA plates [33].

### 2.6. Confocal Laser Scanning Microscopy Analysis of Cell Viability

The 48 h *L. monocytogenes* biofilms were prepared in 35 mm glass-bottom confocal dishes as described in Section 2.5. The biofilms were then washed three times with physiological saline, followed by the addition of 500 μL of NPs at different concentrations and 500 μL of PBS solution to achieve concentrations of 4 MIC, 2 MIC, and MIC. The incubation was continued statically at 37 °C for an additional 12 h. Subsequently, 1.5 μL of SYTO 9 and PI staining solutions were mixed into 1 mL of physiological saline so that the final concentration was 5 mg/mL. The biofilms were washed three times with physiological saline, and the mixed staining solution was added. The samples were incubated in the dark for 15 min. After staining, the biofilms were washed three times with physiological saline to remove excess dye and then observed using CLSM to examine the morphology of *L. monocytogenes* biofilms. Observations were made using a 40× objective lens. The excitation/emission maxima for SYTO 9 were approximately 480/500 nm, and for PI, they were 490/635 nm. Z-axis scanning was performed at a thickness of 0.22 μm to obtain stacked planar images of the biofilms.

### 2.7. Scanning Electron Microscopy Analysis of Biofilms

Biofilm samples treated with different concentrations of NPs were prepared according to the method described in Section 2.3. After washing three times with physiological saline, the samples were fixed with 2.5% glutaraldehyde at 4 °C in the dark for 12 h. The fixed samples were then dehydrated using a graded ethanol series (30%, 40%, 50%, 60%, 70%, 80%, 90%, 95%, and 100%), with each dehydration step lasting 10 min. Subsequently, the samples were dried in an oven, sputter-coated with gold, and observed using SEM (Hitachi, TM 3000, Tokyo, Japan). Images were captured at magnifications of 2000× and 5000×.

### 2.8. Transmission Electron Microscopy Analysis of Cell Morphology

*L. monocytogenes* biofilms were formed on PU samples according to the method described in Section 2.3. In a new 24-well plate, 500 μL of DNase-CS-N and 500 μL of PBS solution were added to achieve a concentration of 2 MIC. After 48 h incubation, the PU samples were washed three times with physiological saline and then transferred to the prepared 24-well plate for an additional 12 h incubation at 37 °C. The DNase-CS-N-treated PU samples were washed three times with physiological saline and then transferred to centrifuge tubes containing 5 mL of physiological saline. The samples were subjected to ultrasonication at 40 kW for 3 min, followed by vigorous vortexing for 30 s. The detached *L. monocytogenes* biofilms cell suspensions were centrifuged at 8000 rpm for 10 min at 4 °C and then fixed with 2.5% glutaraldehyde at 4 °C in the dark for 12 h. Subsequently, the samples were stained with 3% (*w*/*w*) phosphotungstic acid solution for 15 min and air-dried before being observed using TEM (Tecnai 12, Philips, Albuquerque, NM, USA).

### 2.9. Effect of DNase-CS-N on Intracellular Material Leakage of Bacteria Within L. monocytogenes Biofilms

The 48 h *L. monocytogenes* biofilms on PU coupons were washed three times with physiological saline as described in Section 2.3 and then transferred into a new 15 mL centrifuge tube. The tubes were filled with 500 μL of DNase-CS-N and 500 μL of PBS solution, achieving concentrations of 4 MIC, 2 MIC, and MIC. Then, the samples were incubated at 37 °C for 12 h. Subsequently, detached *L. monocytogenes* biofilms cell suspension was obtained as described in Section 2.3. For determination of nucleic acid and protein leakage, the biofilm cell suspension was directly centrifuged at 10,000 rpm for 10 min at 4 °C. The supernatant was then analyzed using a microplate reader to measure the OD_260_, and the protein content was determined using a bicinchoninic acid (BCA) protein assay kit (Nanjing Jiancheng Bioengineering Institute, Nanjing, China). The OD_260_ measurement provides a direct indication of the nucleic acid concentration, often serving as a proxy for the amount of DNA in a sample since these biomacromolecules absorb this particular wavelength of light. Subsequently, to accurately determine the protein content within the supernatant, a BCA protein assay kit was employed. The working solution was prepared according to the kit and mixed with the sample in a 96-well plate; distilled water was used as a blank control instead of the sample, and the protein standard solution served as the standard group for calculating protein concentration. Then, it was incubated at 37 °C for 30 min, and the absorption at 562 nm wavelength was determined.

### 2.10. Statistical Analysis

All data were analyzed in triplicate, and data were expressed as mean ± standard deviation (S.D). A one-way analysis of variance (ANOVA) was conducted using Duncan’s multiple comparison test, and SPSS 16.0 software was performed to compare the differences between the results. The significance level *p* was set at 0.05.

## 3. Results and Discussion

### 3.1. Effect of NPs on Bacterial Cell Population in L. monocytogenes Biofilms Formation

**Figure 1:**
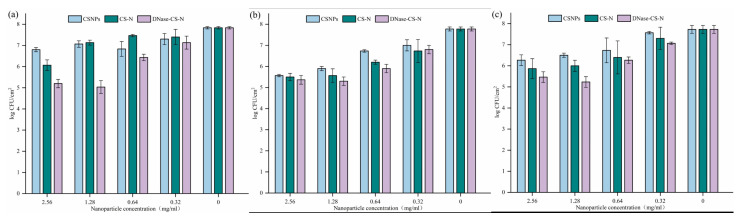
Reduction of viable cells counts of *L. monocytogenes* biofilm formation by nanoparticle treatment (CSNPs: chitosan nanoparticles, CS-N: nisin-loaded chitosan nanoparticles, DNase-CS-N: chitosan nanoparticles loaded with nisin and DNase I): (**a**) stainless steel; (**b**) polyurethane; (**c**) polystyrene.

The MIC of three NPs (CSNPs, CS-N, and DNase-CS-N) was previously determined to be 1.28 mg/mL [26]. The inhibitory activity of NPs against *L. monocytogenes* biofilm bacterial cell population at 2 MIC, MIC, 1/2 MIC, and 1/4 MIC are shown in Figure 1.

Overall, it was obvious that NPs had a concentration-dependent effect on bacterial cell population inhibition. When the concentration was at 1/4 MIC (0.32 mg/mL), all three NPs could decrease the biofilm cell counts to 7.0 log CFU/cm^2^ in contrast to 7.8 log CFU/cm^2^ in the control group. At a concentration of 1/2 MIC (0.64 mg/mL), DNase-CS-N could reduce the biofilm cell counts to 5.9~6.3 CFU/cm^2^ on three surfaces, reaching about a 99% inhibition rate. When the concentration increased to MIC, DNase-CS-N reduced the biofilm cell counts to 5.0~5.3 log CFU/cm^2^, which was significantly lower than those obtained in CSNPs and CS-N treatment (5.9~7.1 log CFU/cm^2^ and 5.6~7.1 log CFU/cm^2^, respectively). However, the inhibitory activity of NPs did not improve when the concentration increased to 2 MIC.

In general, there was no significant difference in the inhibitory effect of NPs for *L. monocytogenes* biofilm formation on three surfaces, with one exception. The biofilm population of *L. monocytogenes* on the SS surface was 7.1 log CFU/cm^2^ with CSNPs and CS-N treatment, which were significantly higher than that on PS (6.5 log CFU/cm^2^ and 6.0 log CFU/cm^2^) and PU (5.9 log CFU/cm^2^ and 5.6 log CFU/cm^2^), respectively.

CSNPs have been previously applied as the nanocarrier for the antimicrobial peptide LL37 to improve antibacterial activity against *S. aureus* and inhibit 68% biofilm formation [34]. Patel et al. [35] also reported enhanced efficacy of DNase grafting on NPs to inhibit biofilm formation of *P. aeruginosa*. This might be explained by the presence of DNase I within the NPs, which might alter the microbial surface adhesion and biofilm production by degrading the eDNA [36].

### 3.2. The Effect of NPs on Cell Motility of L. monocytogenes

**Figure 2:**
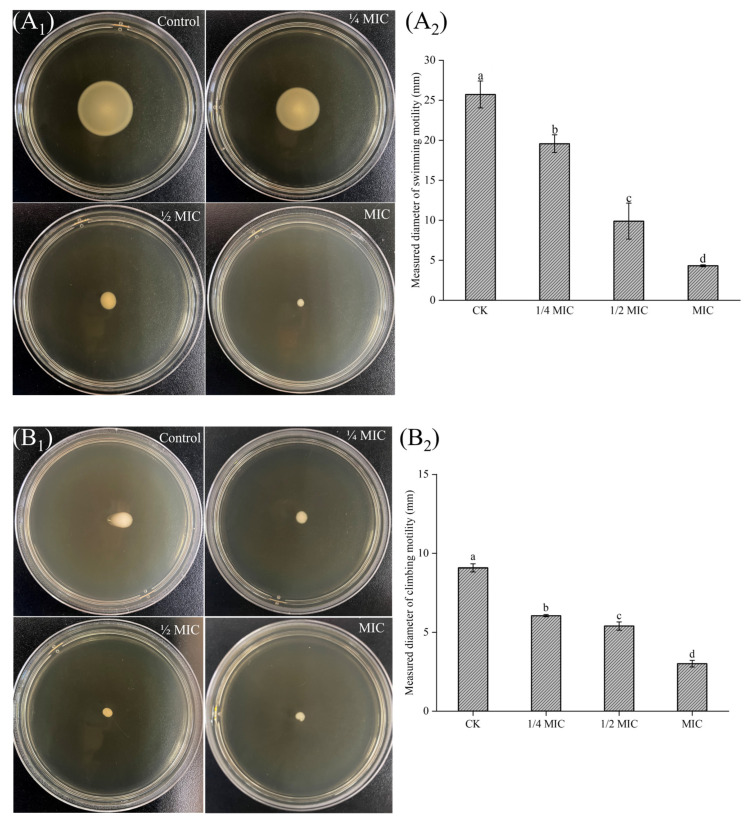
The impact of DNase-CS-N (chitosan nanoparticles loaded with nisin and DNase I on motility of *L. monocytogenes*, (**A_1_**) swimming motility; (**A_2_**) measured diameter of swimming motility; (**B_1_**) swarming mobility; (**B_2_**) measured diameter of swarming motility. Different letters represent a significant difference (*p* < 0.05).

Flagellar motility plays a role in the attachment and biofilm formation of *L. monocytogenes* [37]. The motility of *L. monocytogenes* is temperature-dependent. Motility is effective at temperatures below 30 °C because the major flagellar protein FlaA is highly expressed [38]. Thus, we evaluated the effect of NPs on the motility of *L. monocytogenes* at 30 °C (Figure 2). The control group indicated that *L. monocytogenes* exhibits motility, forming swimming zones with a diameter of 25.7 mm and swarming zones with a diameter of 9.1 mm, respectively. As the concentration of DNase-CS-N increased, the motility of the strain was significantly decreased. For instance, the swimming zone diameter of *L. monocytogenes* was reduced to only 4.3 mm when exposed to DNase-CS-N at MIC, and the swarming zone diameter decreased to 3.0 mm. In accordance with our results, the CSNPs with antibiofilm activity at sub-MIC concentrations could also inhibit the swimming and swarming motility of *P. aeruginosa* [39,40]. Combined with previous biofilm inhibition assays, the inhibitory effect on cell motility of DNase-CS-N might contribute to its antibiofilm activity.

### 3.3. The Effect of NPs on Slime Production of L. monocytogenes

**Figure 3:**
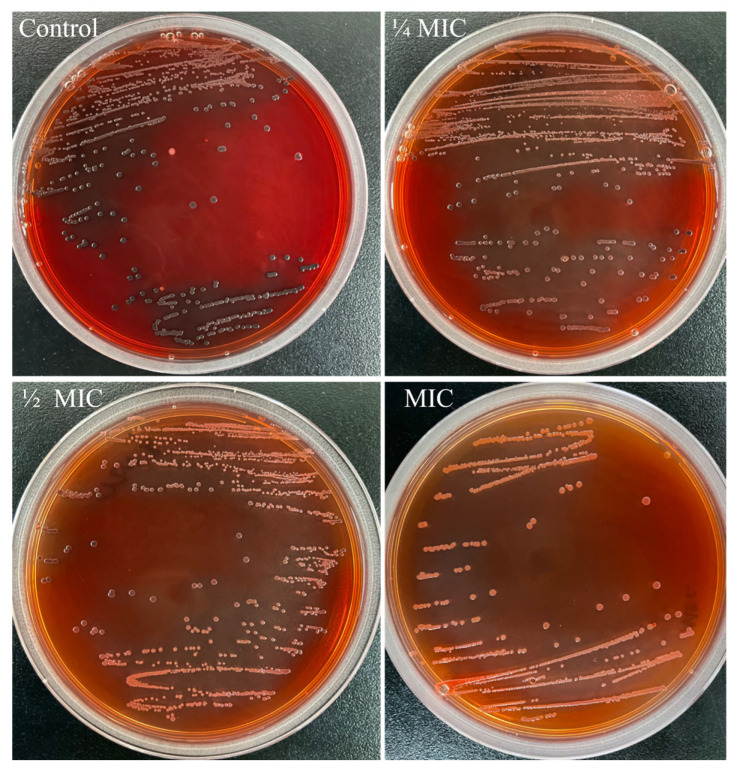
The level of slime production by *L. monocytogenes* treated with DNase-CS-N (chitosan nanoparticles loaded with nisin and DNase I) on CRA (Congo Red Agar) plates.

The slime in nature is the extracellular polysaccharide, which is essential for biofilm formation [31]. Thus, inhibition of slime could indirectly reduce biofilm formation. The CRA plates can be used to assess slime production of *L. monocytogenes* strains, with the positive strains forming black colonies and the negative strains forming red or unchanged color colonies [41]. As shown in Figure 3, the control plate contained substances identified as black colonies, suggesting that the *L. monocytogenes* 57,034 strain used in this study is a slime-producing strain. With increasing concentrations of DNase-CS-N, the number of black colonies gradually decreased, indicating a decrease in slime production. Similarly, Jayasankari et al. [42] constructed jacalin copper sulfide NPs, which could inhibit biofilm formation and slime synthesis of *S. aureus*. These results indicate that DNase-CS-N could reduce slime production in *L. monocytogenes*, thereby inhibiting biofilm formation.

### 3.4. Effect of NPs on Bacterial Cell Population in Preformed L. monocytogenes Biofilms

**Figure 4:**
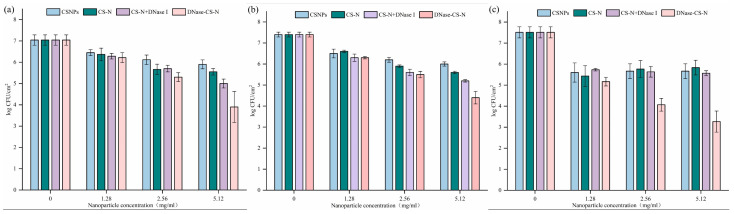
Reduction of viable cell counts in preformed *L. monocytogenes* biofilms by nanoparticle treatment (CSNPs: chitosan nanoparticles, CS-N: nisin-loaded chitosan nanoparticles, DNase-CS-N: chitosan nanoparticles loaded with nisin and DNase I), (**a**): stainless steel, (**b**): polyurethane, (**c**): polystyrene.

The results presented in Figure 4 demonstrate the reduction effect of the biofilm cell population treated with different NPs on food contact surfaces. On SS coupons, the maximum log reduction in biofilm cell density ranged from 1.1 log CFU/cm^2^ (CSNPs) to 3.3 log CFU/cm^2^ (DNase-CS-N). Similarly, the maximum reduction in PU materials was about 1.7 log CFU/cm^2^ (CSNPs) to 3.0 log CFU/cm^2^ (DNase-CS-N). The elimination effect on the PS surface is relatively the best, with a maximum of 4.2 log CFU/cm^2^ (DNase-CS-N). Park et al. [43] treated food-borne pathogens attached to SS and PU with a combination of food-grade compounds. There was no significant difference in the bactericidal efficacy at 0.25% concentration, both of which could immediately inhibit the survival of *E. coli* and *L. monocytogenes* after treatment.

Overall, DNase-CS-N was the most effective treatment across all surfaces, outperforming CS-N + DNase I, CS-N, and CSNPs in eliminating bacterial cell populations in preformed *L. monocytogenes* biofilms. This indicates that the incorporation of DNase I into NPs augmented the degradation effect of biofilm EPS and its antibiofilm activity compared to free DNase I without grafting on NPs, possibly related to the improved penetration effect of NPs with small size. Similar to our study, Patel et al. [35] reported that DNase I functionalized CSNPs loaded with ciprofloxacin demonstrated enhanced biofilm inhibition and dispersion efficiency against *P. aeruginosa* biofilms compared to the NPs without functionalization with DNase I. Baelo et al. [44] also prepared DNase I functionalized polylysine-coated poly (lactic-co-glycolic acid) NPs loaded with ciprofloxacin, which inhibited 95% biofilm formation and eliminated over 99.8% of pre-formed *P. aeruginosa* biofilms. It is well known that DNase I could degrade the eDNA and disassemble biofilm structure, which might improve the penetration of NPs and improve the antimicrobial activity of nisin.

### 3.5. Penetration of Biofilms by DNase-CS-N

**Figure 5:**
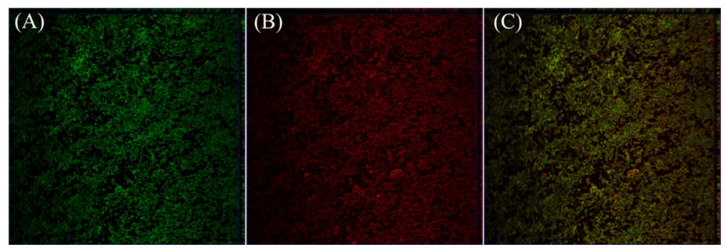
Penetration of biofilms by DNase-CS-N (chitosan nanoparticles loaded with nisin and DNase I). (**A**): SYTO 9 labeled biofilms (green); (**B**): RBITC-DNase-CS-N (red) penetration into biofilms; (**C**): overlap.

As shown in Figure 5, RBITC-DNase-CS-N (red) can be observed on both the surface and interior of the biofilms. Intense red fluorescence was observed throughout the biofilms from the top to the bottom, indicating that DNase-CS-N penetrated into the biofilm matrix. Polymeric nanoparticle carriers have been demonstrated to deliver antimicrobial peptides to enhance antibacterial efficacy [45]. Many biofilm components are negatively charged, such as negatively charged sugar polymers and eDNA [46]. Thus, the positively charged DNase-CS-N can interact with negatively charged biofilm components and microbial cells [47]. Our cationic NPs could easily penetrate into biofilms and bind to anionic biofilm components and the surfaces of microbial cells. Therefore, the positively charged DNase-CS-N can deliver nisin and release it within the biofilms, thereby directly acting on the cells within biofilms.

### 3.6. The Impact of NPs on the Spatial Structure of L. monocytogenes Biofilms

**Figure 6:**
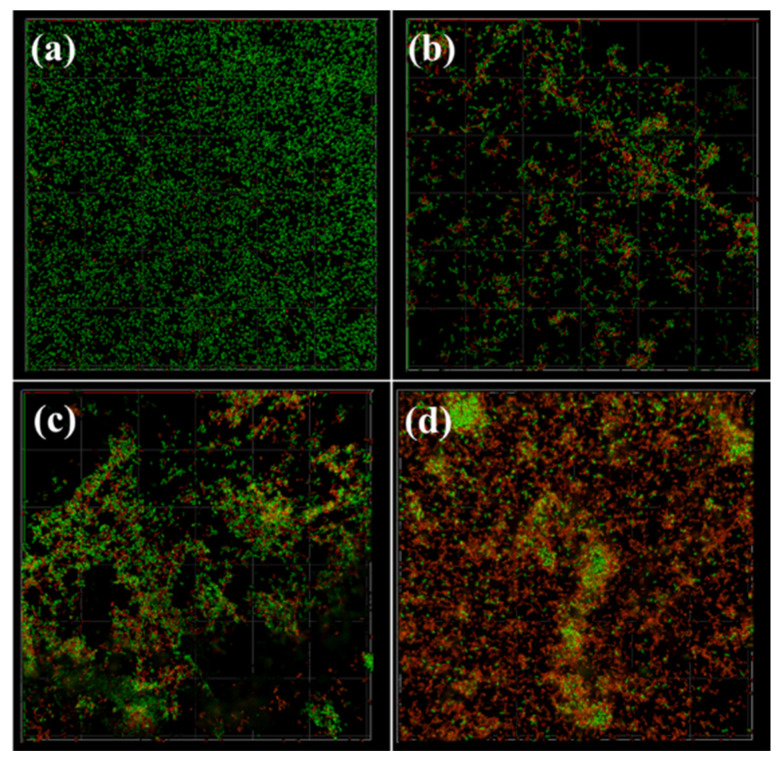
CLSM images of *L. monocytogenes* biofilms after treatment by nanoparticles; live cells are green (SYTO 9), and dead cells are red (PI); (**a**): control, (**b**): 2 MIC CSNPs (chitosan nanoparticles), (**c**): 2 MIC CS-N (nisin-loaded chitosan nanoparticles), (**d**): 2 MIC DNase-CS-N (chitosan nanoparticles loaded with nisin and DNase I).

To evaluate the effect of spatial structure on *L. monocytogenes* biofilms, the preformed *L. monocytogenes* biofilms were exposed to three NPs at 2 MIC, and then the biofilms were subjected to CLSM (Figure 6). For the control group, *L. monocytogenes* formed biofilms with dense cellular carpet-like structures uniformly distributed across the entire surface (Figure 6a). Many green clusters (live cells) were observed, with almost no red (dead cells) fluorescence, indicating a mature biofilm structure with multiple layers of viable cells. After treatment with 2 MIC concentrations of CSNPs and CS-N (Figure 6b,c), the number of dead cells significantly increased compared with control. However, a considerable number of live cells was still observed. As shown in Figure 6d, treatment of DNase-CS-N at 2 MIC resulted in a significantly higher number of dead cells compared to CSNPs and CS-N, indicating that DNase-CS-N leads to a better biofilm elimination effect, resulting in more cell death.

**Figure 7:**
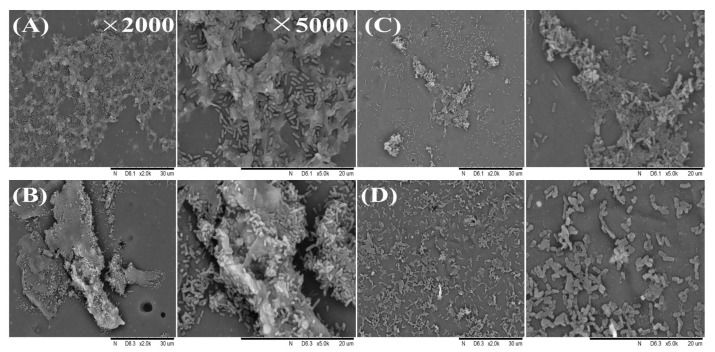
SEM images of *L. monocytogenes* biofilms after treatment by nanoparticles, (**A**): control, (**B**): CSNPs (chitosan nanoparticles), (**C**): CS-N (nisin-loaded chitosan nanoparticles), (**D**): DNase-CS-N (chitosan nanoparticles loaded with nisin and DNase I).

*L. monocytogenes* biofilms treated with different NPs at 2 MIC were analyzed through SEM imaging in order to provide a more intuitive observation of biofilms’ spatial organization. For the control group (Figure 7A), tightly arranged cells were encapsulated by layers of EPS matrix, forming densely packed biofilms with intact rod-shaped cell structures. Following treatment with CSNPs (Figure 7B), the biofilm structure almost remained intact, similar to the control group. In contrast, after CS-N treatment (Figure 7C), a significant portion of the biofilms was evidently disrupted, resulting in loose and sparse cell clusters embedded with EPS. After DNase-CS-N treatment (Figure 7D), the structure of the biofilm was severely disrupted, with bacterial cells scattered on the surface. Additionally, some bacterial cells showed incomplete structures and varied morphologies. This indicates that DNase-CS-N treatment led to severe destruction of mature *L. monocytogenes* biofilm structures, which is consistent with previous plate count and CLSM findings, further confirming the ability of DNase-CS-N to destroy mature biofilms. Similar to our findings, Lin et al. [48] loaded DNase I and glucose oxidase onto CSNPs and found that the dual-species biofilm structure of *S. enterica* and *L. monocytogenes* was disintegrated, which indicates the synergistic antibiofilm activity of NPs. Tan et al. [49] demonstrated that CSNPs loaded with oxacillin and DNase I could reduce biofilm thickness and the number of viable cells of *S. aureus* more effectively compared with the nanoparticle without DNase I. eDNA is an important component of *L. monocytogenes* biofilms and is, therefore, an attractive target for biofilm control. DNase I has been previously used to induce dispersal of *L. monocytogenes* biofilms [50], and it has been combined with other antimicrobials for biofilm inhibition [51]. In this study, the DNase-CS-N could combine the degradation effect of DNase I and the antimicrobial activity of nisin, achieving a synergistic effect on *L. monocytogenes* biofilm elimination.

### 3.7. The Impact of DNase-CS-N on Cell Morphology of L. monocytogenes

**Figure 8:**
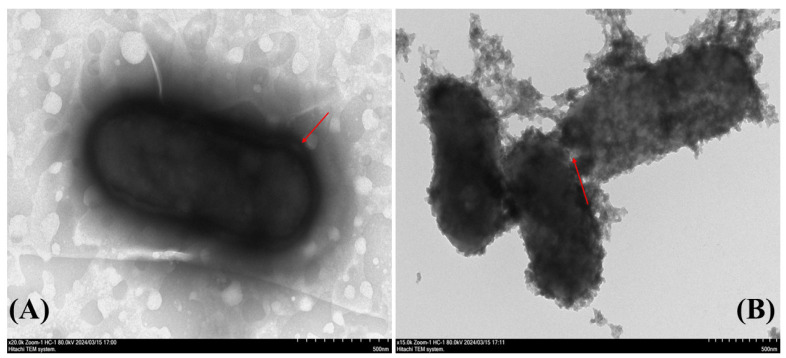
TEM image of *L. monocytogenes* (**A**): control, (**B**) after treatment by DNase CS-N (chitosan nanoparticles loaded with nisin and DNase I). (as shown by the arrows on the left, untreated bacterial cells possess an intact phospholipid bilayer structure and a smooth surface, while DNase-CS-N treated cells exhibit wrinkled surroundings, rough cell surfaces, and incomplete phospholipid bilayers, along with leakage of intracellular macromolecules).

The morphological changes in *L. monocytogenes* cells before and after DNase-CS-N treatment are depicted in Figure 8. The TEM images clearly show that untreated bacterial cells possess an intact phospholipid bilayer structure and a smooth surface (as indicated by the arrow in the left figure). In contrast, cells treated with DNase-CS-N exhibit wrinkled surroundings, rough cell surfaces, and incomplete phospholipid bilayers, along with leakage of intracellular macromolecules. These observations are consistent with the findings of Alebouyeh et al. [52], who reported that *L. monocytogenes* cells treated with nisin lost their original shape, appearing twisted and irregular with small surface pores (as indicated by the arrow in the right figure). In addition to nisin, chitosan is also known to have antimicrobial activity against most bacteria. According to Benabbou et al. [53], chitosan at 20–100 kD could exert its antibacterial effect by forming a thin layer on the cell surface, thereby blocking the entry of essential nutrients and causing cell death. In this study, the chitosan used had a molecular weight of approximately 100 kDa, and a thin layer was observed on the bacterial surface in Figure 8. These findings suggest that DNase-CS-N exhibited a bactericidal effect, resulting in severe cell damage.

**Figure 9:**
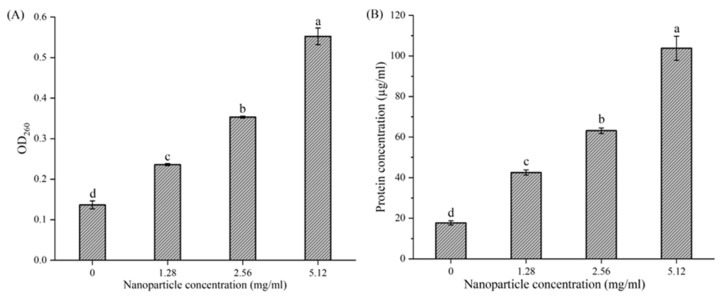
The effect of DNase CS-N (chitosan nanoparticles loaded with nisin and DNase I) on extracellular nucleic acid level (**A**) and protein concentration (**B**). Different letters represent a significant difference (*p* < 0.05).

The integrity of *L. monocytogenes* biofilm cells was also evaluated by measuring the leakage of nucleic acid and protein, which are essential intracellular biomacromolecules. With increasing concentrations of DNase-CS-N, the levels of intracellular nucleic acids and proteins increase accordingly (Figure 9). At 4 MIC, the level of nucleic acid is 3.9 times higher than that in the control group, and the concentration of protein is 6.5 times higher than that in the control group. These results indicate that DNase-CS-N damaged the cell integrity of *L. monocytogenes* biofilm cells and caused a significant leakage of intracellular nucleic acids and proteins, which is corroborated by previous TEM images. Consistent with these results, Lan et al. [54] also found the chitosan-grafted caffeic acid exhibited antibacterial activity against *Pseudomonas fluorescens* by destroying the somatic bacterial cells, resulting in the leakage of cellular contents and death by lysis.

The possible mechanisms by which DNase-CS-N eliminates biofilms in this study are as follows: First, due to its nanoscale structure, DNase-CS-N can better penetrate the biofilm matrix, thereby imparting its inherent antibacterial properties [26,55]. Secondly, due to its positive charge, DNase-CS-N can electrostatically interact with the negatively charged biofilm components and bacterial cell membranes, affecting membrane permeability and causing leakage of cytoplasmic contents, leading to cell death [34,56]. Additionally, DNase I on the nanoparticle surface can degrade eDNA, disrupting the biofilm extracellular matrix and directly delivering high concentrations of nisin to biofilm cells [35,49]. The materials selected for nanoparticle preparation in this study, including CS, TPP, nisin, DNase I, EDC, and NHS, are all safe and non-toxic green materials that have been widely used in the food industry. In summary, DNase-CS-N more effectively disrupts *L. monocytogenes* biofilms by simultaneously targeting the biofilm matrix and biofilm cells. Therefore, these results indicate that NPs targeting both eDNA and biofilm cells show great potential in effectively eradicating biofilms and could be potentially applied as anti-*L. monocytogenes* biofilm agents in the food industry.

## 4. Conclusions

The inhibitory effect of DNase-CS-N on cell viability, motility, and spatial structures of *L. monocytogenes* biofilm was investigated in this study. At sub-MICs, DNase-CS-N could effectively inhibit the biofilm formation of *L. monocytogenes*, possibly related to its ability to reduce cell motility and slime production. Compared with CS-N + DNase I treatment, CS-N and CSNPs, DNase-CS-N at 2 MIC and 4 MIC showed the stronger ability to eliminate preformed biofilms, achieving a 3–4 log reduction in the biofilm population. Moreover, the spatial structures of *L. monocytogenes* biofilms were severely disrupted, and the cell morphology was also damaged by the leakage of cellular substances. These findings offer promise for the potential application of DNase-CS-N in controlling *L. monocytogenes* in food processing environments. However, it is necessary to further investigate the anti-biofilm mechanism of DNase-CS-N and assess its efficacy against mixed-species biofilms in complex food processing systems.

## Data Availability

The original contributions presented in the study are included in the article, further inquiries can be directed to the corresponding author.
